# Ultrahigh adsorption and singlet-oxygen mediated degradation for efficient synergetic removal of bisphenol A by a stable zirconium-porphyrin metal-organic framework

**DOI:** 10.1038/s41598-017-06194-z

**Published:** 2017-07-24

**Authors:** Ai-Na Meng, Ling-Xiao Chaihu, Huan-Huan Chen, Zhi-Yuan Gu

**Affiliations:** Jiangsu Key Laboratory of Biofunctional Materials, Jiangsu Collaborative Innovation Center of Biomedical Functional Materials, School of Chemistry and Materials Science, Nanjing Normal University, Nanjing, 210023 P. R. China

## Abstract

Bisphenol A (BPA), one of 23 most important endocrine disrupting chemicals, was efficiently removed and sequentially photodegraded by a zirconium-porphyrin metal–organic framework (MOF) catalyst under visible light for water treatment. Well control of photodegradation allows the kinetic separation of adsorption step and photodegradation step. Ultrahigh adsorption uptake of 487.69 ± 8.37 mg g^−1^ is observed, while efficient photodegradation could be observed within 20 min at the rate of 0.004 mg min^−1^. The synergetic effect boosts the photocatalytic efficiency and confirms that the catalysis happens inside the MOF pores other than in the solution phase. Furthermore, the mechanism was elucidated by diverse control experiments, such as in the conditions of ^1^O_2_ scavenger, in darkness and with the changes of light sensitizing ligands. It confirmed that BPA was oxidized by the ^1^O_2_ which was generated from porphyrin ligand within MOFs under visible-light. The excellent reusability and wide range of suitable pH range make the Zr-porphyrin MOFs practical for the photocatalytic water treatment processes.

## Introduction

Metal–organic frameworks (MOFs) with tunable pore size and modifiable pore surfaces have been extensively studied due to their promising applications in gas storage, separation, sensing, catalysis and environmental applications^1–21^. Photocatalysis is a convenient way to utilize the energy of sunlight or artificial illumination to achieve chemical transformation in the green earth and renewable energy projects^22–25^. Efficient photocatalysis with MOFs have been used for water splitting^23–26^ and CO_2_ reduction with the photogeneration of free radicals and electrons^26–28^. Recently, a different type of photocatalysis, the photogeneration of singlet oxygen (^1^O_2_) from light-sensitizing MOF attracted immense research efforts due to the potential applications in photodynamic therapy (PDT) and degradation of chemical warfare agent^29–37^.

Singlet oxygen (^1^O_2_) is the lowest excited state oxygen molecule, which can be obtained by the energy transfer from an excited triplet state of a photosensitizer to ground-state molecular oxygen (^3^O_2_)^38–42^. ^1^O_2_ is more eco-friendly and efficient than free radicals in the reaction with certain classes of organic compounds (e.g., furans and phenols)^43, 44^. Although the ^1^O_2_ is very promising in the degradation of contaminants from waste water^45–47^, there exists no report over the photodegradation of BPA by MOFs for organic pollutant treatment based on ^1^O_2_.

Porphyrin derivatives with high photosensitizing efficiency have been widely used in the production of singlet oxygen^48–55^. To explore the applications in aqueous solutions, zirconium based tetrakis (4-carboxyphenyl)-porphyrin (TCPP) MOFs have been synthesized^37, 56^. Although zirconium-porphyrin with exceptional stability has been applied in several heterogeneous catalysis^26, 28, 37, 57, 58^, the mechanism is still obscure. It is hard to kinetically distinguish the adsorption step and catalysis step within the normal MOF catalysis due to the simultaneous existence of permanent porosity and internal catalytic sites^59^. The controllable degradation via singlet oxygen under light irradiation in MOFs^37, 60^ kinetically separate the adsorption and catalysis processes. It gives a great opportunity to investigate their synergy effect, which has never been explored.

Bisphenol A (BPA) is the most widely used and industrially produced bisphenols, which is regarded as one of the most important 23 endocrine disrupting chemicals (EDCs) by World Health Organization (WHO)^61^. BPA is a good candidate molecule because its molecular kinetic diameter is smaller than the pore size of MOFs^62–64^. At the same time BPA is possibly degraded by singlet oxygen^65–67^.

Herein, to facilitate the adsorption, diffusion and photodegradation of BPA molecules, we have selected a mesoporous zirconium-porphyrin MOF PCN-222 (also named as MOF-545 or MMPF-6) with 3.7 nm permanent channels^28, 37, 68^, which is also highly stable in aqueous solution and capable of ^1^O_2_ generation under visible-light irradiation. The investigation of synergetic effects between stepwise adsorption and photodegradation could be achieved sequentially by adsorption under dark and degradation under visible light. Ultrahigh adsorption uptake of 487.69 ± 8.37 mg g^−1^ is observed, while efficient photodegradation could finish within 10 min. The synergetic effect boosts the photocatalytic efficiency as well as confirms that the catalysis happens inside the MOF pores other than in the solution phase. Furthermore, the mechanism was elucidated by the control experiments with ^1^O_2_ scavenger, darkness and changes of light sensitizing ligand. It confirmed that BPA was oxidized by the ^1^O_2_ which was generated by TCPP ligand in MOFs under visible-light irradiation. The reusability, stability and possible reaction pathway about photocatalytic degradation of BPA by PCN-222 were explored, we found PCN-222 had excellent reusability and wide range of suitable pH range.

## Results and Discussion

### Characterization of the PCN-222

**Figure 1:**
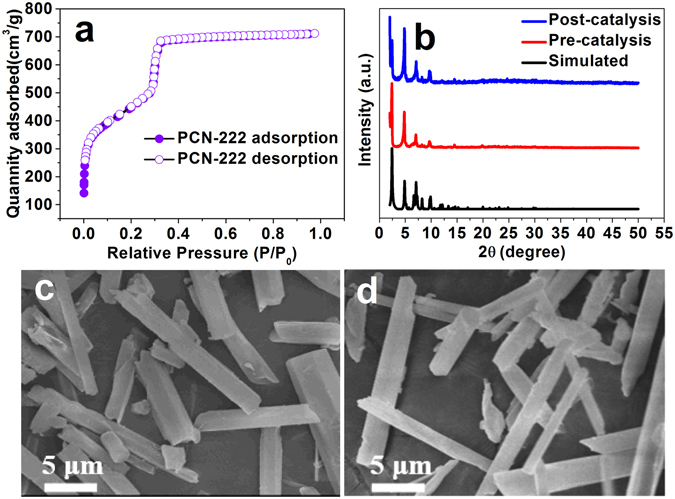
(**a**) The nitrogen adsorption-desorption isotherms of PCN-222 at 77 K. (**b**) PXRD patterns of PCN-222 for the simulated, pre-catalysis and post-catalysis samples. SEM images of (**c**) pre-catalysis PCN-222 and (**d**) PCN-222 after 5-cycles catalysis.

The PCN-222 was synthesized according to the existing methods, and well characterized by XRD, N_2_ adsorption–desorption experiment and SEM (Fig. 1). The XRD measurements demonstrated that the synthesized PCN-222 experimental XRD pattern was consistent with the simulation diagram, which proved PCN-222 was successfully prepared. The size of single crystalline rod-like PCN-222 was in micro-meter scale (Fig. 1c). The SEM image of PCN-222 after catalysis was shown in Fig. 1d, confirming that the MOFs kept intact morphology even after 5-cycles catalysis. The prepared PCN-222 gave a BET surface area of 1914 m^2^ g^−1^ with a pore volume of 1.03 cm^3^ g^−1^. The pore size distribution of PCN-222 estimated by the Barrett–Joyner–Halenda method gave a pore diameter of 3.59 nm (Figure S1). The isostructural MOF with a different light sensitizing ligand, namely PCN-222-Fe(III)Cl was also successfully prepared and well characterized for further exploration of ^1^O_2_ generation mechanism (Figure S2).

### Adsorption for BPA on PCN-222: Thermodynamics, Kinetics and suitable pH range

**Figure 2:**
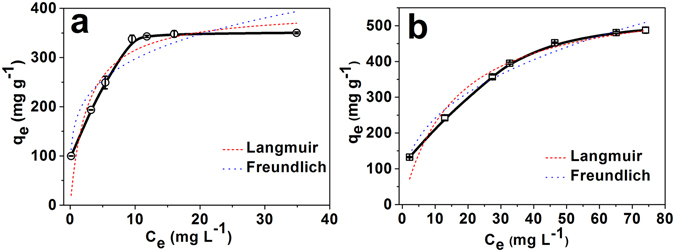
Adsorption isotherms of (**a**) 100 ppm BPA and (**b**) 250 ppm BPA at 25 °C and pH 8.0 for 60 min. Langmuir and Freundlich models were fitted to isotherms, respectively.

To fully reveal the potential of the adsorption of BPA by PCN-222, the static adsorption of BPA with high concentrations were performed. Small amounts of organic phase of ethanol were added to water to increase BPA solubility as well as to evaluate the solvent effect in real applications. Therefore, the adsorption isotherms for BPA on PCN-222 were plotted to demonstrate the sorption capacities with two different BPA solutions, which are BPA of 100 ppm in water/ethanol (249:1, v/v) solution and BPA of 250 ppm in water/ethanol (245:5, v/v) solution, respectively (Fig. 2).

The maximum adsorption capacity of PCN-222 (487.69 ± 8.37 mg g^−1^) is the highest record compared to the other sorbents in the previous reports (Table S1)^62^. It is because PCN-222 has a three-dimensional structure with high surface area and ultra-large one-dimensional channels in 3.6 nm diameter^28^. The uptake of 487.69 ± 8.37 mg g^−1^ reveals the adsorption information in molecular level that 15 BPA molecules were adsorbed per unit cell, corresponding to one meso-channel unit (3.7 nm i.d.) and two micro-channel units (1.1 nm i.d.). Stoichiometrically, the 15 BPA molecules were adsorbed to 6 photo-sensitizing porphyrin ligands through van der Waals interactions for further catalysis.

To clearly elucidate the thermodynamic adsorption of BPA in PCN-222, Langmuir and Freundlich models were employed to fit the isothermal adsorption plots, respectively. Comparing to the empirical Freundlich isotherm, the theoretical Langmuir isotherm assumes that maximum coverage of the solid surface with a monolayer of the adsorbate molecules (see Supplementary Information Section S5). The fitting results of the Langmuir and Freundlich models are compared and shown in Fig. 2 while the corresponding parameter values are shown in Table S2. For the BPA concentration of 100 ppm, both Langmuir and Freundlich models are not fitting well with the R^2^ values of 0.815 and 0.856, respectively. Meanwhile, for the high BPA concentration of 250 ppm, R^2^ values for the Langmuir and Freundlich sorption isotherm were 0.947 and 0.976, respectively. The fitting results were in accordance to the structural properties of PCN-222. Basically, there are two adsorption sites in PCN-222 for BPA molecules, meso-channels (3.7 nm i.d.) and micro-channels (1.1 nm i.d.). In this case, Freundlich isotherm describes better in the multisite adsorption isotherm for rough surfaces. However, the micro-channels are only big enough for the adsorption of single BPA molecule (1.1 nm × 0.6 nm) per unit cell. Therefore, the meso-channel is the primary adsorption site and possibly adsorbs up to 15 BPA molecules per unit cell, rendering the monolayer adsorption within meso-channels. The current ultrahigh adsorption uptake record could be broken by the increase of organic solvent concentrations. However, considering the synergetic effect, monolayer adsorption type is good to further need of the photocatalysis. Therefore, no higher than 250 ppm concentrations were selected for BPA in the all following experiments.

The adsorption removal efficiency for BPA was also calculated. It is impossible to reach high removal efficiency and adsorption uptake at the same time. More adsorbents or low BPA concentrations will result in high removal efficiency. To make a fair comparison, 1.0 mg PCN-222 was added to 1.0 mL BPA aqueous solution with different concentrations. It showed up to 99.9% removal efficiency at 100 ppm BPA as well as 86.8% at 250 ppm, respectively. As the environmental concentrations for BPA were 0.002–269 ppb^69^, the pre-enrichment is difficult step in the practical treatment of the environmental samples. The high adsorption capacity and removal efficiency indicate ultrahigh equilibrium constant *K* which will be very practical in the enrichment of BPA with low concentrations.

**Figure 3:**
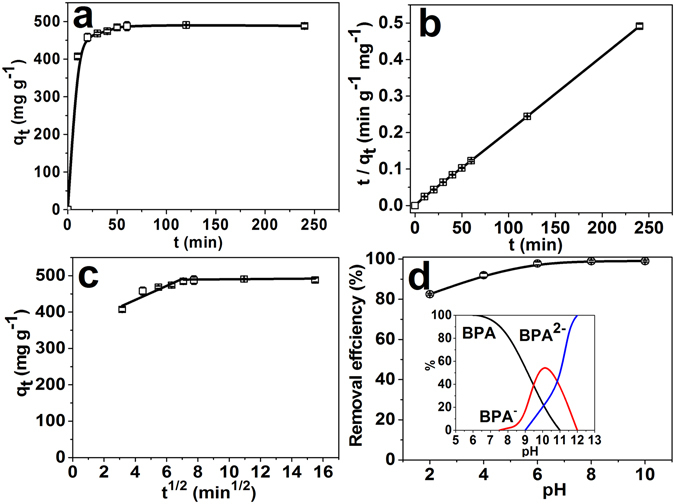
(**a**) The kinetic curve for the adsorption of BPA on PCN-222 at 250 ppm of BPA at 25 °C and pH 8.0. (**b**) Data fitting with pseudo-second-order kinetics model. (**c**) Data fitting with intra-particle diffusion model. (**d**) Effect of pH on the adsorption of BPA on PCN-222 at 25 °C (Sorption conditions: C_0_ = 250 ppm, V = 1.0 mL, and m_MOF_ = 1.0 mg). Insert shows the effect of pH on distribution of different BPA chemical forms.

The adsorption kinetics of BPA on PCN-222 were also evaluated and illustrated in Fig. 3a. The sorption rate of BPA on PCN-222 changed with time. In the first 10 minutes, the adsorption amount of BPA increased significantly and the adsorption capacity of 407.14 ± 6.08 mg g^−1^ was reached. From 10 to 40 minutes, the adsorption amount of BPA increased slowly with final equilibrium at 40 minutes and adsorption capacity of 487.69 ± 8.37 mg g^−1^. To well understand the adsorption kinetics, the pseudo-second-order kinetics model and intra-particle diffusion model were employed to fit kinetic data.

1 $$\mathrm{Pseudo}-\mathrm{second}-\mathrm{order}\,{\rm{kinetic}}\,\mathrm{model}:\,\frac{t}{{q}_{t}}=\frac{1}{{k}_{2}{q}_{e}^{2}}+\frac{t}{{q}_{e}}$$

in which *q*_*e*_ and *q*_*t*_ are the adsorption capacity (mg g^−1^) at equilibrium and at time *t* (min), respectively, while *k*_2_ is the rate constant for pseudo-second-order adsorption (g mg^−1^ min^−1^). The dependence of time for the adsorption of BPA on PCN-222 can be well fitted by a general pseudo-second-order kinetic model with R^2^ of 0.9999 rather than other models (Fig. 3b, Table S3 and Figure S4). The theoretical saturation adsorption capacity of 490.1 ± 1.8 mg g^−1^ calculated by pseudo-second-order kinetic model is consistent with the experimental value of 487.69 ± 8.37 mg g^−1^. So BPA adsorption behaviour by PCN-222 is mainly physical adsorption, and its adsorption rate is affected by the concentration of BPA.

In order to further analyze the dynamic diffusion mechanism, the intra-particle diffusion model was used, as the following equation:

2 $${q}_{t}={k}_{i}\times {t}^{\frac{1}{2}}+I$$

where *q*_t_ (mg g^−1^) is the adsorption capacity at time *t*, *k*_*i*_ (mg g^−1^ min^−0.5^) is the intra-particle diffusion rate constant, and *I* is the coefficient associated with the thickness of boundary layer. If *I* = 0, then the rate of adsorption is only restricted by intra-particle diffusion for the total adsorption process. The fitting plot of intra-particle diffusion shows two portions of straight lines (Fig. 3c). According to fitting parameters (Table S3), *I* ≠ 0, it indicates that boundary layer diffusion also affects the rate of adsorption while intra-particle diffusion itself was not solely rate-limiting step^70^.

The pH has important influence on the stability of PCN-222 and the chemical forms of BPA in aqueous solution^62^. Figure 3d shows the effect of pH on the adsorption of BPA by PCN-222. The removal efficiency of BPA by PCN-222 increased from 82.5% to 97.8% in the pH range of 2–6, while the removal efficiency remained high removal efficiency in the pH range of 6–10 with highest removal efficiency of 99.3%. The results indicated that PCN-222 has a wide pH range for applications. The removal efficiency was affected in the extreme pH. When the pH was larger than 12, the structure of PCN-222 would be instable and BPA was mostly ionized to divalent anions (BPA^2−^), thus reduced the sorption capacity. At the same time, the sorption capacity decreased in strong acid as the porphyrin in PCN-222 was protonated. Considering the structure of BPA molecules and adsorption capacity of adsorbents, all other experiments were carried out at pH 8.0.

### Visible light photocatalytic degradation of BPA by PCN-222

In order to demonstrate the advantage of synergetic effects between adsorption and photodegradation of BPA in MOFs, the BPA solution (100 ppm) was first adsorbed by MOFs without visible light irradiation for 1 h to ensure the complete adsorption (Figure S5). To fully elucidate the degradation efficiency of BPA by PCN-222 under visible light irradiation, both the BPA concentration in aqueous solution and the residual BPA quantity within PCN-222 were monitored during the catalytic processes. Besides, for comparison, three controlled trials were prepared with the following procedures, which the samples were treated without illumination, replacing PCN-222 with PCN-222-Fe(III)Cl and adding singlet oxygen scavenger.

**Figure 4:**
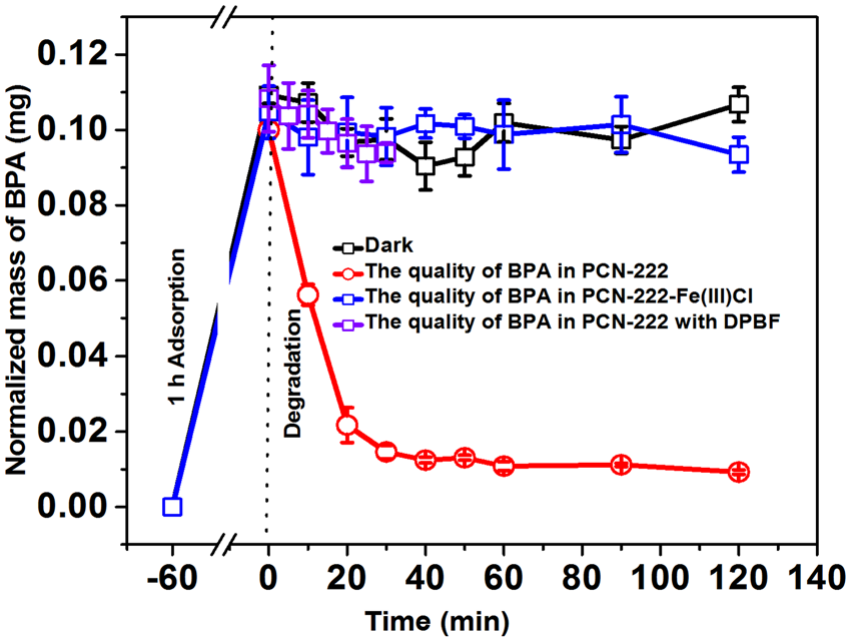
The synergetic adsorption and degradation of BPA with different conditions: PCN-222 in dark (black curve); PCN-222 under visible light (red curve); another isostructural MOF with a different sensitizing ligand PCN-222-Fe(III)Cl under visible light (blue curve) and PCN-222 with ^1^O_2_ scavenger DPBF (purple curve) at 25 °C. BPA initial concentration:100 ppm; the quantity of MOFs: 1.0 mg; pH: 8.0; solution volume: 1.0 mL.

The BPA concentration in aqueous solution was monitored during the adsorption and catalytic processes. Due to high adsorption efficiency of MOFs (over 99%), the residual concentration of BPA in aqueous solution approached to 0.0006 mg after adsorption and all of the remaining BPA was adsorbed in porous channel of PCN-222. (Figure S6). The significant photodegradation of BPA was observed mainly on PCN-222 adsorbent by ethanol elution and GC-MS monitoring (red curve in Fig. 4). It was found that from 0 to 20 min, the mass of BPA in the PCN-222 channel decreased sharply, demonstrating a pseudo zero-order kinetic model with the degradation rate constant of 0.004 ± 0.0002 mg min^−1^ (see Supplementary Information Section S6). It is worth noting that our catalytic system follows pseudo zero-order kinetic while other materials obeys first-order kinetic^71–75^. It was mainly because of the high BPA concentrations and mesopore confinement, which originate from the pre-enrichment of BPA in PCN-222 that 15 BPA molecules were stoichiometrically pre-adsorbed to 6 photo-sensitizing porphyrin ligands along the mesoporous channels. The degradation rate gradually slowed down after 20 min.

The intermediate products in the process of photocatalytic degradation were screened using GC-MS analysis. In addition to the peak of BPA at m/z 213, one intermediate ion was discovered with m/z 108. The retention time of 5.158 min for the intermediate product with m/z 108 was significantly different from BPA (13.296 min) on HP-5 GC column. The intermediate product was identified as 1,4-benzoquinone by NIST database (Figure S7). To further confirm the intermediate and the catalytic pathway, the mass of BPA and 1,4-benzoquinone in the solution phase and adsorbed by PCN-222 were monitored with different irradiation time, respectively (Figure S8). The equilibrium concentration of the intermediate in aqueous solution reached the maximum after 20 min-irradiation, then it gradually decreased and tended to be constant. From Figure S8b, it can be seen that the trend of intermediate concentration in PCN-222 was similar to aqueous solution (Figure S8a) and the amount of intermediate in aqueous solution was higher than that extraction from PCN-222. This suggests that the intermediate of 1,4-benzoquinone formed in PCN-222 channels in the process of photocatalytic degradation and diffused from the channel into the aqueous solution.

To evaluate the effect of visible light irradiation, catalysis under dark condition was performed as control experiment and the results were shown as black curve in Fig. 4. No significant catalysis was observed under dark conditions within 120 min. The quantity of BPA in PCN-222 under dark conditions was 0.1 ± 0.007 mg and from 100 ppm BPA aqueous solution (1 mL), showing good recovery. The significant errors come from the extraction procedures of BPA from heterogeneous PCN-222 before GC analysis. Normalized data were shown to justify the comparison.

The porphyrinic MOFs are capable of generating ^1^O_2_ under photo irradiation for the oxidation of chemical warfare agent^37^. For PCN-222, the degradation of BPA could be effective due to the direct oxidation by ^1^O_2_ in the presence of visible light. The observation of only *m*/*z* 108 (1,4-benzoquinone) and no hydroxylated products were observed in the reaction process which may suggest that the reaction is not based on other free radicals^73^. Based on the results above, the process of photodegradation of BPA by PCN-222 is the direct oxidation by ^1^O_2_, which is generated from PCN-222 under visible light irradiation (Figure S5). As previously reported, the final products of ^1^O_2_ oxidation of BPA were CO_2_ and H_2_O^71^. It is highly practical in the water treatment for BPA oxidation by ^1^O_2_ since it is green and the final products would be CO_2_ and H_2_O.

### Explore the catalytic mechanism by visible light photocatalytic degradation of BPA with PCN-222-Fe(III)Cl and TCPP ligand

Porphyrin as the photosensitizer have been extensively employed for ^1^O_2_ generation. In order to verify the catalytic mechanism, instead of TCPP, we synthesized another porphyrin ligand, iron(III) porphyrin chloride to form MOF PCN-222-Fe(III)Cl, which works as Fenton’s reagent for Type I catalysis pathway by generating hydroxyl radical^53^. We used PCN-222-Fe(III)Cl as catalyst to establish photocatalytic degradation of BPA under the same conditions as performed with PCN-222. No significant catalytic degradation of BPA under visible light irradiation was observed on PCN-222-Fe(III)Cl (blue curve in Fig. 4), at the same time, no intermediate (*m*/*z* 108, 1,4-benzoquinone) was detected during the photocatalytic process (Figure S9). To further confirm the role of porphyrin, it was found that BPA can also be rapidly degraded by only TCPP ligand (Figure S10), which proved that the degradation of BPA mainly occurs on the site of TCPP ligand in PCN-222. It is reasonable that PCN-222 with 3-D structure has better performance of high adsorption capacity and good stability comparing with TCPP ligand.

### Explore the catalytic mechanism with ^1^O_2_ scavenger

**Figure 5:**
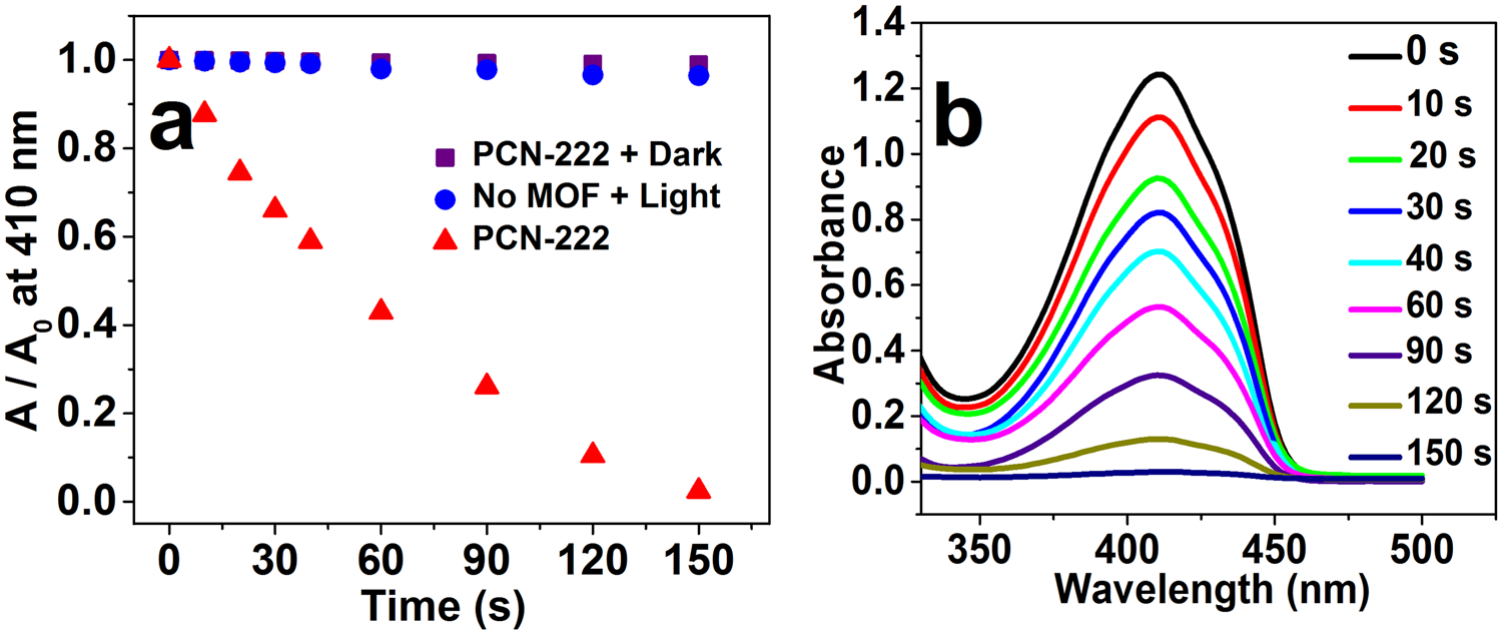
(**a**) Absorbance decay of the reacted DPBF solution. (**b**) The corresponding spectra in the presence of PCN-222. In every experiment, 2 mg PCN-222 was added to 10 mL the initial concentration of 60 µM DPBF acetonitrile solution (with O_2_ bubbled). Visible light irradiation conditions: wavelength range of 480–700 nm.

To further confirm the ^1^O_2_ generation ability of TCPP in PCN-222, we choose 1,3-diphenylisobenzofuran (DPBF) as the ^1^O_2_ scavenger^38^. The absorbance of DPBF at λ = 410 nm would decrease when DPBF was oxidized by ^1^O_2_, which acted as a specific indicator of ^1^O_2_ generation. As shown in Fig. 5, the absorbance of DPBF (60 µM) in acetonitrile with PCN-222 at λ = 410 nm was rapidly decreased within 150 seconds under visible light irradiation, while the absorbance was kept without PCN-222 under the same irradiation as a control experiment. The control experiment in dark was also performed. It indicates PCN-222 is an excellent photosensitizer for generating ^1^O_2_. To well explore the reaction mechanism and further confirm the effect of ^1^O_2_, DPBF was added to eliminate ^1^O_2_ during the photodegradation. To guarantee the successful removal of ^1^O_2_, the 100 ppm BPA aqueous solution was first bubbled with nitrogen for 30 minutes. Then 10 µL DPBF acetonitrile solution of 1 mol L^−1^ was added to the catalytic solution after adsorption equilibrium with PCN-222. It can be observed from purple curve in Fig. 4 that the quality of BPA with the adding of DPBF was almost unchanged within 30 min under visible light irradiation compared to that without adding of DPBF. No intermediate products were detected during the first five minute (Figure S11). The photocatalytic degradation with DPBF was monitored for only 30 min because DPBF is unstable and sensitive to the external factors such as UV light and heat. It indicated that ^1^O_2_ generated by PCN-222 under visible light was shown to be an effective degradation reagent for BPA.

### Stability of PCN-222 under visible light irradiation

The stability of PCN-222 was investigated by recycling experiment. After the end of each adsorption-photocatalytic experiment, PCN-222 was recovered by centrifugation and then applied to the next adsorption-photocatalytic degradation experiment. After five cycles, the removal efficiency of PCN-222 still keeps at 99.2% for the adsorption-degradation of BPA (Figure S12). The PCN-222 after five cycles still keeps its rod-like shape and micrometer-scale size (Fig. 1d). The results indicated that PCN-222 has a stable and efficient catalytic performance for removal of BPA by combined action of adsorption and degradation under visible light irradiation.

## Conclusions

In this work, Zr-porphyrin MOF PCN-222 was utilized as a dual function material for the adsorption and photocatalytic degradation of BPA under visible light irradiation. PCN-222 exhibits ultrahigh removal efficiency of BPA from aqueous phase and the maximum adsorption capacity is up to 487.69 ± 8.37 mg g^−1^. In addition, PCN-222 maintained the high adsorption capacity as well as chemical stability with wide pH range of 2–10. Under visible light irradiation, PCN-222 was demonstrated to generate ^1^O_2_, which presents excellent degradation performance towards BPA. More importantly, the permanent porosities and the high surface areas of PCN-222 can enhance the enrichment of BPA, thereby accelerating the catalytic process. We believe that the recyclable and environment-friendly PCN-222 with such superior performance will have a broad application in the removal and degradation of water pollutants.

## Methods

### Synthesis of PCN-222

For a typical preparation of PCN-222, ZrCl_4_ (50 mg), H_2_TCPP (50 mg) and benzoic acid (2700 mg) in 8 mL of DEF were ultrasonically dissolved in a 20 mL Teflon-lined autoclave. The mixture was heated at 120 °C for 48 h and then 130 °C for 24 h. After cooling down to room temperature, purple needle shaped crystals were harvested by centrifugation (12000 rpm) after 3 min. For the activation procedure, the obtained as-synthesized PCN-222 was suspended in a solution of 1.5 mL of 4 M HCl in 100 mL DMF and stirred at 120 °C for 12 h. Afterwards, the sample was centrifuged and washed for three times sequentially with DMF and acetone. Then the sample was dispersed in 100 mL acetone for 24 h and the sample was isolated by centrifugation (12000 rpm). Finally, the sample was dried in vacuum for 12 h at 120 °C.

### Sorption assay

For exploring the adsorption ability of PCN-222 for BPA, we prepared 100 ppm and 250 ppm BPA aqueous solutions which were prepared the volume ratio of ethanol and water was 1:249 and 5:245, respectively. The adsorption experiments were carried out by added different amounts of PCN-222 in a serious of 1.0 mL BPA solution (100 ppm and 250 ppm) in 2.0 mL screw vial at room temperature in darkness. At given time intervals, in order to determine BPA concentration in solution phase, samples were filtered with 25 mm × 0.22 μm filter membranes. Then 1.0 mL dichloromethane was added to the residual BPA solution and well mixed to extract the BPA. Finally, 1 μL of BPA in dichloromethane was injected to GC-MS for quantitative analysis with SIM mode of m/z 213 for BPA. The amount of BPA adsorbed on the adsorbent, *q*_*e*_ (mg g^−1^) at equilibrium and *q*_*t*_ (mg g^−1^) at time *t*, respectively were calculated according to the following formula:

3 $${q}_{e}=\frac{({C}_{0}-{C}_{e})}{m}V$$

4 $${q}_{t}=\frac{({C}_{0}-{C}_{t})}{m}V$$

in which *C*_*0*_ and *C*_*e*_ are the initial and the equilibrium concentrations of BPA (mg L^−1^), respectively, *C*_*t*_ (mg L^−1^) is the concentration of BPA at time *t*, *V* (L) is the volume of the BPA solution, and *m* (g) is the quantity of adsorbent.

5 $${\rm{Langmuir}}\,{\rm{model}}:{q}_{e}=\frac{b{q}_{m}{C}_{e}}{1+b{C}_{e}}$$

6 $${\rm{Freundlich}}\,{\rm{model}}:{q}_{e}={K}_{F}\times {c}_{e}^{\frac{1}{{\rm{n}}}}$$

in which *q*_*e*_ is the equilibrium adsorption capacity (mg g^−1^) while *C*_*e*_ is the equilibrium concentrations of BPA (mg L^−1^). *q*_*m*_ is the Langmuir monolayer maximum sorption capacity (mg g^−1^) while *b* is the Langmuir constant (L mg^−1^). *K*_*F*_ (mg g^−1^) and 1/n are the capacity and intensity of sorption Freundlich constants, respectively.

### Degradation experiments

The photocatalytic degradation of BPA by PCN-222 under visible light irradiation experiment was carried out in 2 mL screw vials under the light intensity of 109.2 mW·cm^−2^. After PCN-222 absorb BPA reaching the adsorption equilibrium, the samples were exposed to visible light, which was provided by a Xenon lamp (75 mV, 15 A) filtered from 400 nm to 700 nm. To fully elucidate the degradation efficiency, both the BPA concentration in aqueous solution and the residual BPA quantity within PCN-222 were monitored at certain time intervals after irradiation. For the determination of BPA concentrations in solution, the method is same as which used in sorption assay. For the quantitative analysis of BPA adsorbed in MOF, the solids were first filtered through 25 mm × 0.22 *μ*m membrane, 0.5 mL of ethanol was used to wash twice for MOFs in the filter. Finally, 1 μL of BPA in ethanol was injected to GC-MS for quantitative analysis with SIM mode of m/z 213 for BPA. For the analysis of intermediates of BPA degradation, GC-MS with scan mode was employed. Normalization was performed according to the average recovery rate for the quantitation of BPA washed from MOFs.

## Electronic supplementary material

Supplementary Information
