# Supplementary material for: Ultrahigh adsorption and singlet-oxygen mediated degradation for efficient synergetic removal of bisphenol A by a stable zirconium-porphyrin metal-organic framework
Source: Sci Rep. 2017 Jul 24;7:6297. doi: 10.1038/s41598-017-06194-z (PMC5524690; doi:10.1038/s41598-017-06194-z)
Supplement: Supplementary file 1 — Supplementary Information [file 41598_2017_6194_MOESM1_ESM.pdf]

Supplementary Information

Ultrahigh adsorption and singlet-oxygen mediated degradation for efficient synergetic removal of bisphenol A by a stable zirconium-porphyrin metal-organic framework

Ai-Na Meng, Ling-Xiao Chaihu, Huan-Huan Chen, Zhi-Yuan Gu*

Jiangsu Key Laboratory of Biofunctional Materials, School of Chemistry and Materials Science, Nanjing Normal University, Nanjing 210023

Email: guzhiyuan@njnu.edu.cn, Fax/Phone: +86-25-85891952

Table of Contents

Section S1. **Chemicals and Instrumentation**

Section S2. **Preparation of porphyrin ligand and PCN-222-Fe(III)Cl**

Section S3. **Characterization of PCN-222 and PCN-222-Fe(III)Cl**

Section S4. **GC-MS Conditions and Analysis**

Section S5**. Adsorption Studies**

Section S6. **Catalytic Studies**

Section S1. Chemicals and Instrumentation.

All the chemicals used are at least of analytical grade. N,N-diethylformamide (DEF) and methyl 4-formylbenzoate were purchased from TCI. Co., Ltd. Bisphenol A (BPA, 99%), pyrrole, propionic acid, 1,3-diphenylisobenzofuran (DPBF) and ferrous chloride tetrahydrate ($FeCl_2 \cdot 4H_2O$) were purchased from Aladdin Industrial Inc (Shanghai, China). Zirconium (IV) chloride ($ZrCl_4$) was purchased from J&K Scientific Ltd. (Shanghai, China). N,N-dimethylformamide (DMF), ethanol, ethyl acetate, triethanolamine (THF), chloroform ($CHCl_3$), acetonitrile (C_2H_3N) and benzoic acid were purchased from Sinopharm Chemical Reagent Co., Ltd (Shanghai, China).

Xenon lamp HSX-F300 (Beijing) was employed for the visible light irradiation with 400 nm filter. The lamp conditions were set as 75 mV and 15 A. GC-MS Agilent 7890B/5977A MSD was employed in all the chromatographic separation and mass spectrometric analysis. UV–Vis Hitachi UH5300 was used for kinetically monitoring the reaction between DPBF and singlet oxygen.

Section S2. Preparation of porphyrin ligand and PCN-222-Fe(III)Cl

Synthesis of tetrakis (4-carboxyphenyl) porphyrin (H_2TCPP) ligand

We synthesized the ligand according to the previous report with minor changes.[1] Typically, the mixture of pyrrole (3.0 g, 0.043 mol), methyl *p*-formylbenzoate (6.9 g, 0.042 mol) and propionic acid (100 mL) was added into a 250 mL three-neck flask, and then refluxed for 12 h. After cooling down to the room temperature, the product was washed with ethanol, ethyl acetate and THF sequentially until the elution solution became clear, and then dried in a vacuum oven at 80 ^{o}C. The obtained product of ester TPP-COOMe (1.5 g), KOH (5.25 g, 94 mmol), H_2O (46 mL), THF (46 mL) and MeOH (46 mL) were added into a 250 mL three-neck flask. This mixture was stirred and refluxed for 12 h. After cooling down to room temperature, the homogeneous solution was acidified with 1M HCl until purple solid was precipitated. The purple solid was isolated by the centrifugation (12000 rpm, 5 min], washed with water and dried in vacuum oven at 100 ^{o}C overnight.

Synthesis of [5,10,15,20-Tetrakis(4-carboxyphenyl)porphyrinato]-Fe(III) Chloride (Fe-TCPP-Cl).

We synthesized the ligand according to the previous report.[1] Typically, a solution of ester TPP-COOMe 0.854 g (1.0 mmol) and $FeCl_2 \cdot 4H_2O$ (2.5 g, 12.8 mmol) in 100 mL of DMF was refluxed for 6 h. After the mixture was cooled down to room temperature, 150 mL of H_2O was added. The

resultant precipitate was filtered and washed with plenty of water. The obtained solid was dissolved in $CHCl_3$, then washed three times with 1 M HCl and twice with water. The organic layer was dried over anhydrous magnesium sulfate and evaporated to obtain dark brown crystals. The obtained TPP-COOMe-Fe(III)Cl ester (0.6 g) was stirred in THF (20 mL) and MeOH (20 mL) mixed solvent, to which a solution of KOH (2.104 g, 37.56 mmol) in H_2O (20 mL) was introduced. This mixture was refluxed for 12 h. After cooling down to room temperature, THF and MeOH were evaporated. Additional water was added to the resulting water phase and the mixture was heated until the solid was fully dissolved, then the homogeneous solution was acidified with 1M HCl until no further precipitate was detected. The brown solid was collected by filtration, washed with water and dried in vacuum.

Synthesis of PCN-222-Fe(III)Cl [1]

$ZrCl_4$ (70 mg), Fe-TCPP-Cl ligand (50 mg) and benzoic acid (2700 mg) in 8 mL of DEF were ultrasonically dissolved in a 20 mL Teflon-lined bomb. The mixture was heated in 120 ºC oven for 48 h. After cooling down to room temperature, dark brown needle shaped crystals were harvested by filtration. The activation process for the PCN-222-Fe(III)Cl was as same as which of PCN-222.

Section S3. Characterization of PCN-222 and PCN-222-Fe(III)Cl

The X-ray diffraction (XRD) patterns were recorded on Rigaku D/MAX 2500/PC with Cu Kα radiation from 2° to 50°. The morphology of PCN-222 was collected on a field-emission scanning electron microscope JSM-7600F (JEOL Ltd). N_2 sorption was measured using a Micromeritics ASAP 2050 system at 77 K.

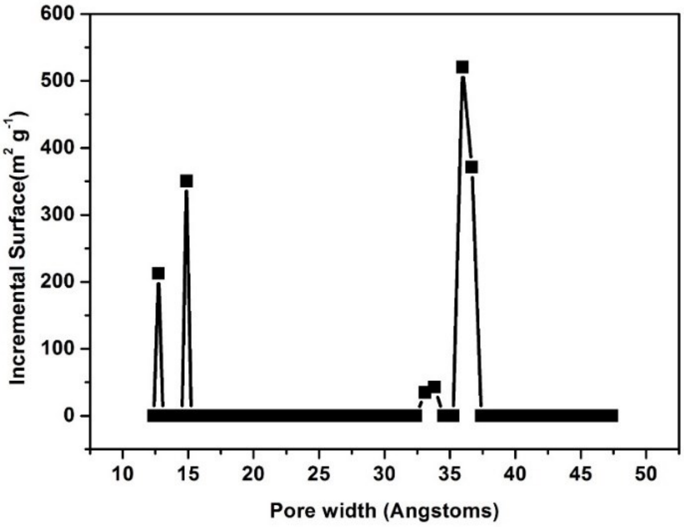

Figure.S1 Barrett–Joyner–Halenda (BJH) pore size distribution for PCN-222.

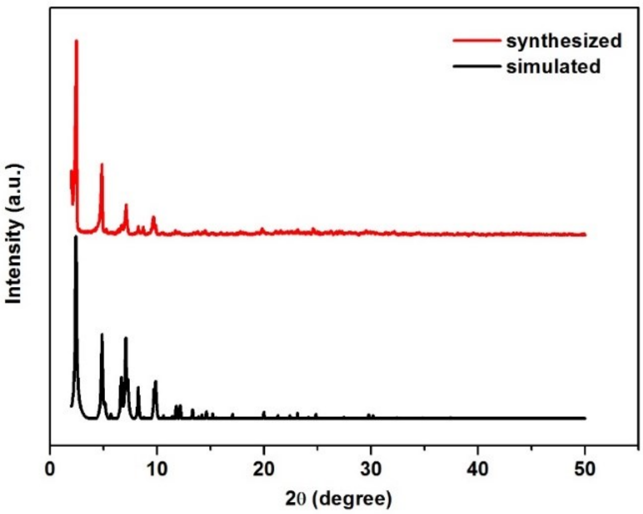

Figure. S2 The simulated (black) and as-synthesized (red) PXRD patterns for PCN-222-Fe(III)Cl.

Section S4. GC-MS Conditions and Analysis

BPA in the reaction solution was analyzed on an Agilent GC-MS, equipped with a fused silica capillary column (30 m × 0.25 mm i.d. × 0.25 μm film thickness). Helium was used as carrier gas at a constant flow of 1 mL min^{-1}. Automatic injection of 1 μL samples was conducted at the split mode and split ratio was 10:1. The injection temperature was 270 °C. The column temperature was programmed as follows: 2 min at 160 °C, 30 °C min^{-1} to 280 °C. BPA was analyzed with electron ionization (EI) source in the selected ion monitoring (SIM) mode at m/z 213. The ion source temperature and the GC-MS transfer line temperature were 230 °C and 280 °C, respectively.

Identification of the intermediates were performed with Scan mode on GC-MS. The column temperature was programmed as follows: 1 min at 50 °C, 10 °C min^{-1} to 150 °C and then 30 °C min^{-1} to 280 °C. Other GC-MS conditions set up as above. The peak with m/z 108 and retention time of 5.158 was discovered during catalysis and marked as intermediate product. Quantitation of the intermediate and reaction kinetics were also investigated.

Quantitation of BPA by standard curves.

Due to the high adsorption efficiency of our MOFs, the quantitation of BPA in low concentration is strongly needed. The BPA concentrations of 0.2, 0.4, 0.8, 1, 2, 20, 30, 40, 50, 60, 80, 90, 100, 110, 120 ppm in ethanol

solution were used to prepare the standard curves where the chromatographic peak area (SIM, m/z 213) was used. One calibration equation could not cover all points. Three calibration curves were used to calculate in the adsorption experiments and catalysis experiments. The standard curves were shown in Figure. S3.

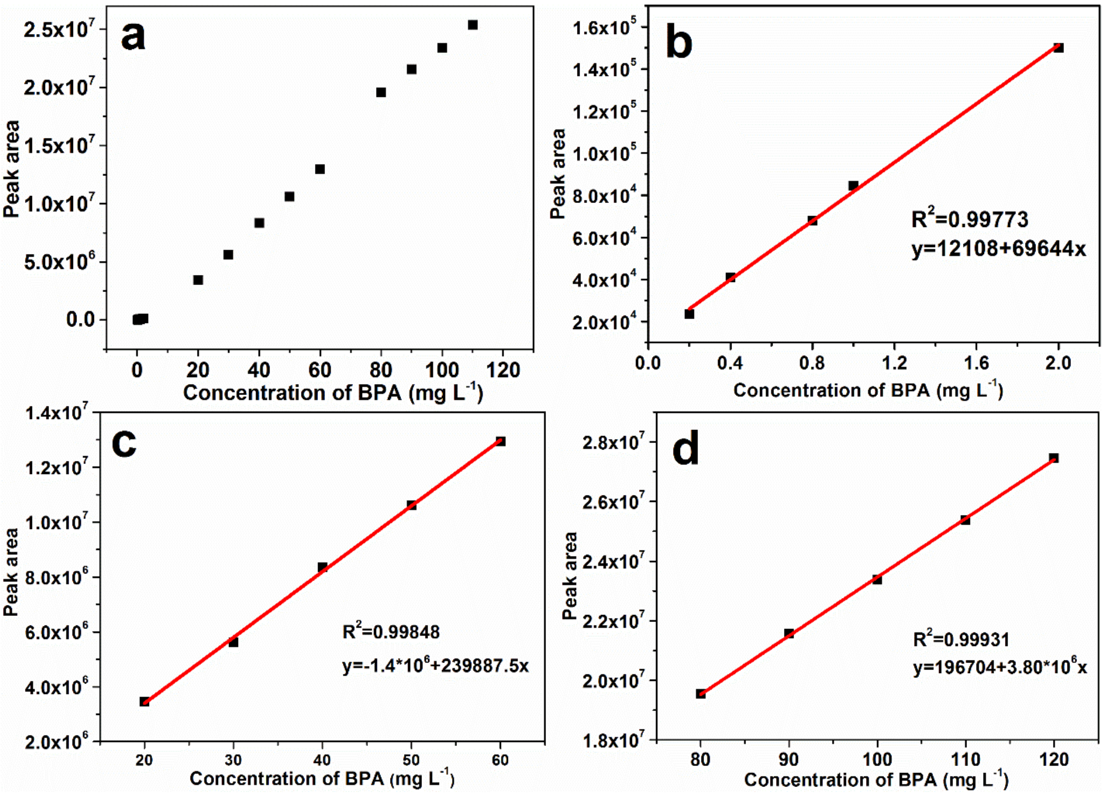

Figure. S3 Data points and standard curves of BPA aqueous solution in different concentration ranges. (a) 0.2-120 ppm; (b) 0.2-2.0 ppm; (c) 20-60 ppm; (d) 80-120 ppm.

Section S5. Adsorption Studies

Pseudo-first-order model : $ln(q_e - q_t) = lnq_e - K_1t$ (1)

in which K_1 (g (mg min)$^{-1}$) is the pseudo-first-order sorption rate constant. q_e (mg g^{-1}) and q_t (mg g^{-1}) are the amount of BPA sorbed per mass of sorbent at equilibrium and at time t, respectively.

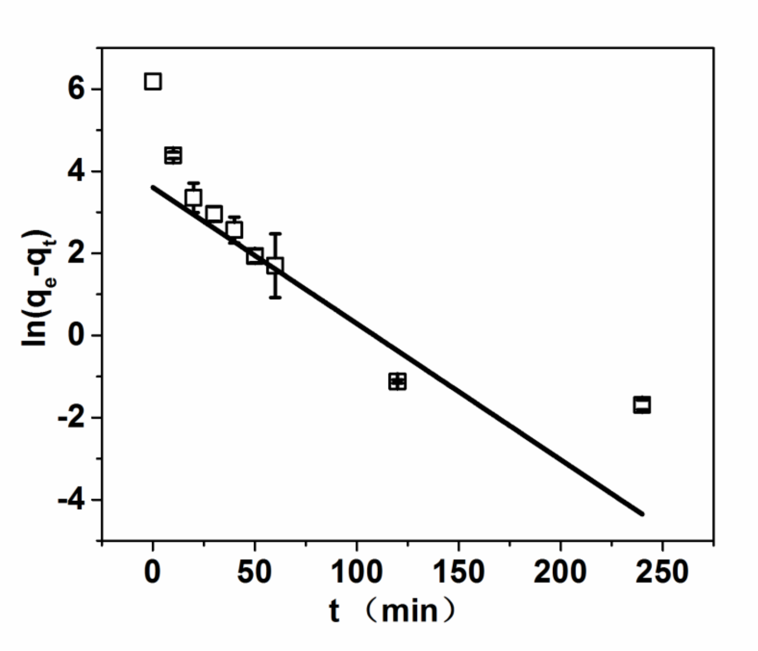

Figure. S4 Data fitting with pseudo-first-order model (Sorption conditions: C_0 = 250 ppm, V = 1.0 mL and m_{MOF} =1.0 mg).

Table S1. Comparison of sorption capacities of BPA on PCN-222 and other sorption materials.

Adsorbents	Maximum sorption capacity (mg g^{-1})	Surface area (m^2 g^{-1})
Modified fibric peat[2]	31.40	0.547
Magnetic molecularly imprinted polymers[3]	142.86	142.90
Organic–inorganic hybrid mesoporous material[4]	351 ± 6.9	750
Hydrophobic zeolite[5]	141	504.5 ± 4.8
Commercial activated carbons[6]	129.6-263.1	1084-1225
MIL-53(Al)[7]	325	931.33
MIL-53(Al)-F127[7]	465	1008.29
PCN-222 (this work)	487.69 ± 8.37	1914

Table S2. Langmuir and Freundlich parameters for the fitted sorption isotherms of bisphenol A.

C_0 (mg L^{-1})	Langmuir			Freundlich		
	q_m (mg g^{-1}) [a]	b (L mg^{-1})	R^2	K_F (mg g^{-1})	1/n	R^2
100	397.6 ± 44.2	0.382	0.815	177.1	0.225	0.856
250	590.8 ± 47.6	0.063	0.947	102.3	0.374	0.976

[a]The results are reported as the mean ± standard deviation

Table S3. Kinetic parameters for the adsorption of BPA on PCN-222 at 25 °C.

C_0 (mg L^{-1})	q_e[a] (mg g^{-1})	pseudo-second-order			intraparticle diffusion model		
		q_e[a] (mg g^{-1})	K_2 (g (mg min)$^{-1}$)	R^2	K_i[b] (mg (g min$^{1/2}$))	I[c] (mg g^{-1})	R^2
250	487.7±8.4	490.1±1.8	0.0020	0.9999	19.28	356.2±21.34	0.8645

[a]The results are reported as the mean ± standard deviation. [b] intraparticle diffusion rate constant for the first linear portion. [c] intercept for the first linear portion.

Section S6. Catalytic Studies

The removal efficiency (R) of BPA was calculated using the following equation: $R = (1 - C/C_0)$ (2)

where C_0 is the initial concentration of BPA and C is the concentration in the aqueous solution.

The photocatalytic degradation of BPA by PCN-222 (pH 8) from 0 to 20 min followed a pseudo-zero-order kinetic model :

$k_{app}t = C_0\text{-}C$ (3)

Where k_{app} is the apparent pseudo-zero-order rate constant, which was determined by plotting $C_0\text{-}C$ versus reaction time t.

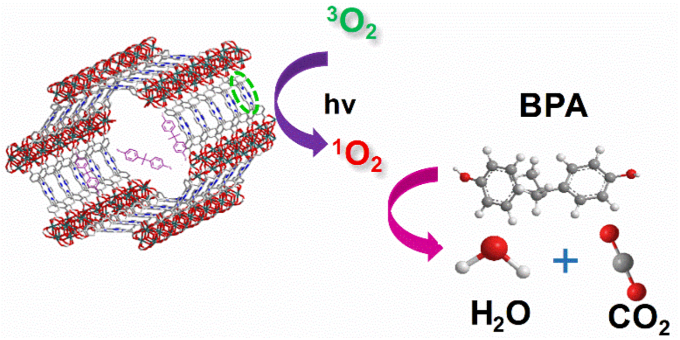

Figure. S5 Proposed mechanism for photocatalytic BPA by PCN-222 under visible-light irradiation.

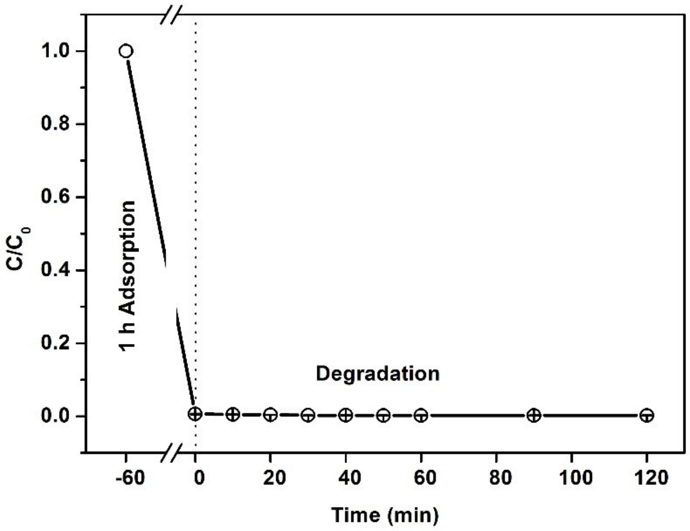

Figure. S6 The concentration of BPA in aqueous solution during the adsorption and degradation.

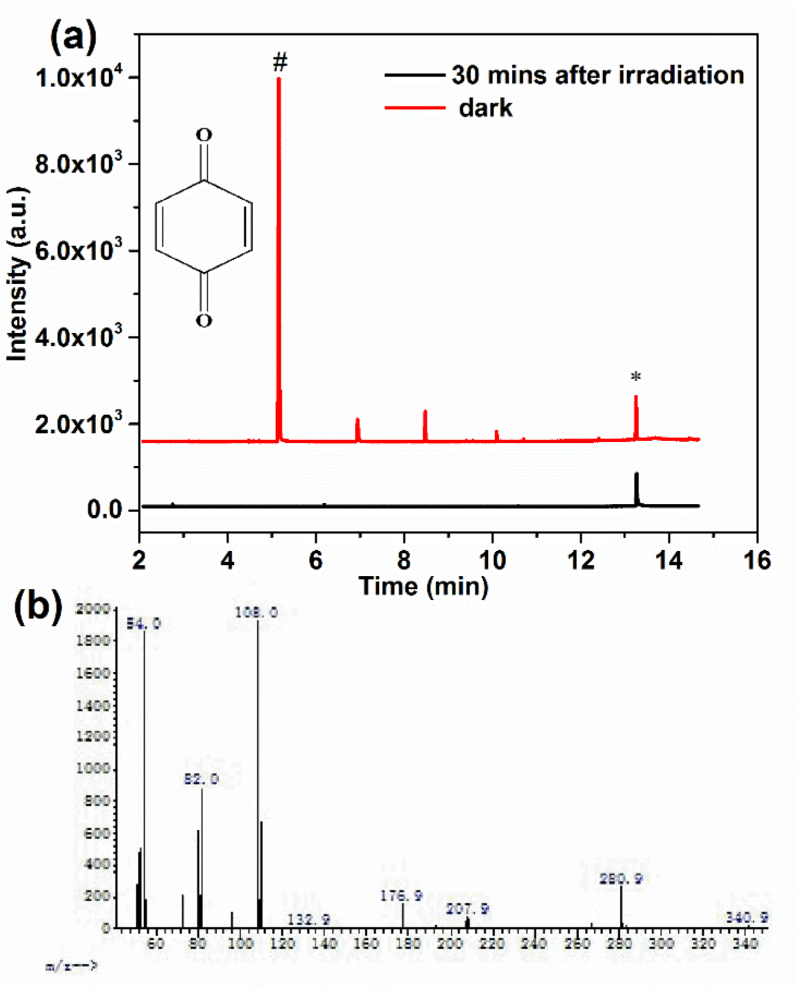

Figure. S7 (a) The comparison of chromatograms for the solution phase under dark and irradiation for 30 min, respectively confirms the photodegradation capability of PCN-222 and elucidate the intermediate product (SIM: m/z of 108 and 213; 1 min at 50 °C, 10 °C min^{-1} to 150 °C and then 30 °C min^{-1} to 280 °C); (b) The mass spectra of the intermediate product which has the retention time of 5.158 min. # represents the intermediate product, * represents BPA. Unmarked peaks represent undesired siloxane peaks from column bleeding.

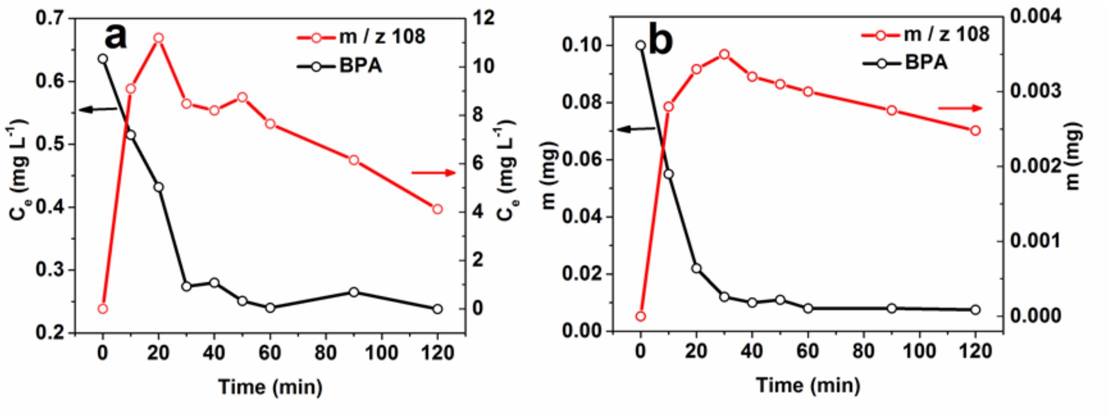

Figure. S8 (a) The concentration of BPA and proposed reaction intermediate (m/z 108) in aqueous solution and (b) the concentration of BPA and proposed reaction intermediate in PCN-222 (BPA initial concentration:100 ppm; the quantity of PCN-222: 1 mg; pH: 8)

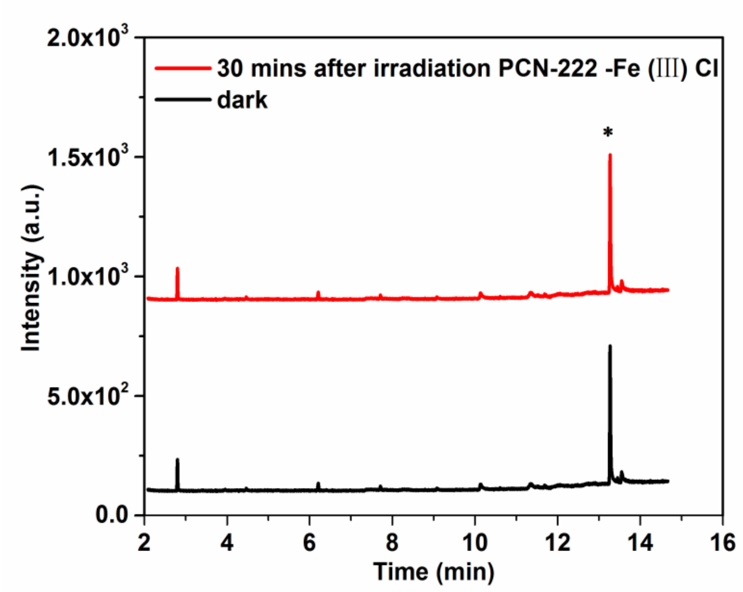

Figure. S9 The comparison of chromatograms for the solution phase under dark and irradiation for 30 min, respectively confirms that PCN-222-Fe(III)-Cl could not perform photodegradation. * represents BPA. (SIM: m/z of 108 and 213; 1 min at 50 °C, 10 °C min^{-1} to 150 °C and then 30 °C min^{-1} to 280 °C)

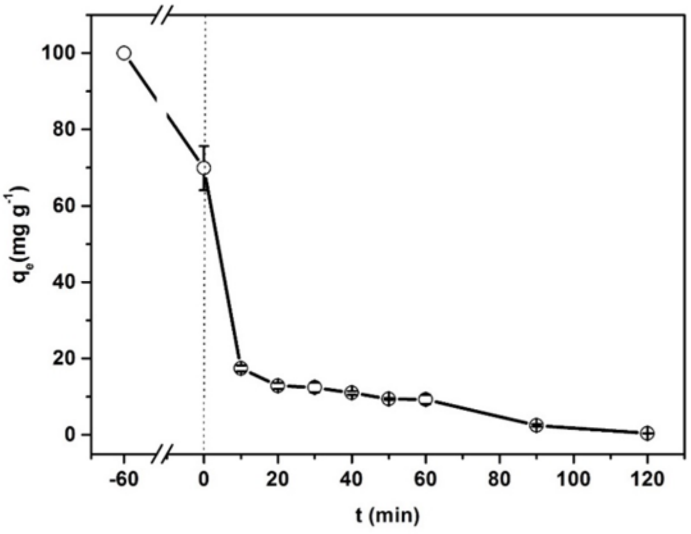

Figure. S10 The concentration of BPA in aqueous solution with the H_2TCPP as catalyst during the adsorption and photodegradation (BPA initial concentration:100 ppm; the quantity of H_2TCPP: 1 mg; pH: 8).

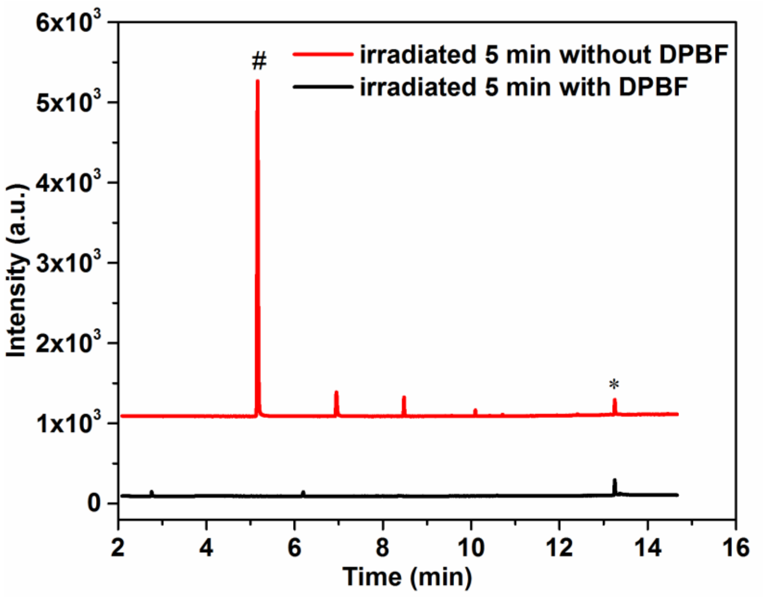

Figure. S11 The comparison of chromatograms for the solution phase under irradiation for 5 min with and without $^1O_2$ scavenger DBPF, respectively to confirm that $^1O_2$ is the key reactant.

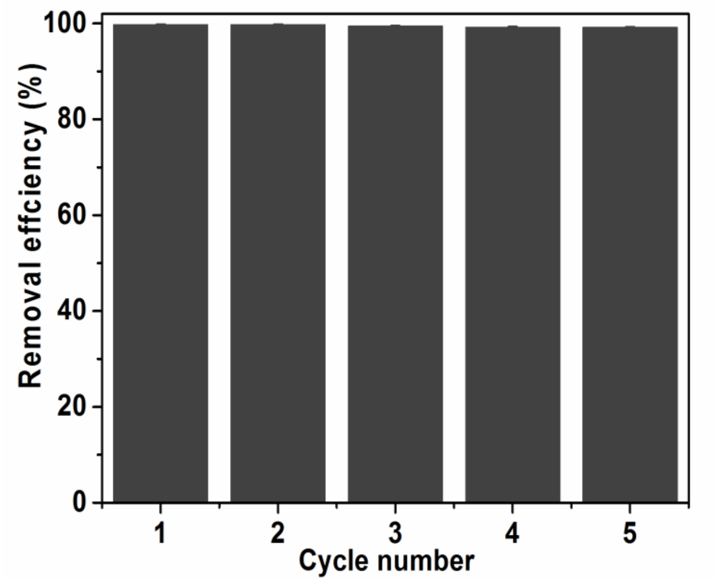

Figure. S12 PCN-222 recycling for the removal of BPA. (BPA initial concentration:100 ppm; the quantity of PCN-222: 1.0 mg; pH: 8.0)

References

1 Feng, D. et al. Zirconium-Metalloporphyrin PCN-222: Mesoporous Metal–Organic Frameworks with Ultrahigh Stability as Biomimetic Catalysts. *Angew. Chem. Int. Ed.* **51,** 10307-10310 (2012).

2 Zhou, Y., Li, N., Zhuang, W. & Wu, X. Vascular endothelial growth factor (VEGF) gene polymorphisms and gastric cancer risk in a Chinese Han population. *Mol. Carcinogen.* **50,** 184-188 (2011).

3 Guo, W. et al. Selective adsorption and separation of BPA from aqueous solution using novel molecularly imprinted polymers based on kaolinite/Fe_3O_4 composites. *Chem. Eng. J.* **171,** 603-611 (2011).

4 Kim, Y.-H., Lee, B., Choo, K. H. & Choi, S. J. Selective adsorption of bisphenol A by organic–inorganic hybrid mesoporous silicas. *Microporous Mesoporous Mater.* **138,** 184-190 (2011).

5 Tsai, W. T., Hsu, H. C., Su, T. Y., Lin, K. Y. & Lin, C. M. Adsorption characteristics of bisphenol-A in aqueous solutions onto hydrophobic zeolite. *J. Colloid Interf. Sci.* **299,** 513-519 (2006).

6 Bautista-Toledo, I., Ferro-Garcia, M. A., Rivera-Utrilla, J., Moreno-Castilla, C. & Vegas Fernandez, F. J. Bisphenol A removal from water by activated carbon. Effects of carbon characteristics and solution chemistry. *Environ. Sci. Technol.* **39,** 6246-6250 (2005).

7 Zhou, M. et al. The removal of bisphenol A from aqueous solutions by MIL-53(Al) and mesostructured MIL-53(Al). *J. Colloid Interf. Sci.* **405,**

157-163 (2013).
